# A facile route to synthesize n-SnO_2_/p-CuFe_2_O_4_ to rapidly degrade toxic methylene blue dye under natural sunlight†

**DOI:** 10.1039/d2ra01690g

**Published:** 2022-06-06

**Authors:** Kaijiao Duan, Tingting Que, Sivasankar Koppala, Ramdas Balan, Budigi Lokesh, Rahul Pillai, Selvaraj David, Parasuraman Karthikeyan, Sangeetha Ramamoorthy, I. C. Lekshmi, Patiya Kemacheevakul, Nagarajan Padmavathy, Sathishkumar Munusamy

**Affiliations:** School of Chemistry and Environment, Yunnan Minzu University Kunming 650505 Yunnan China; Saveetha School of Engineering, Saveetha Institute of Medical and Technical Sciences (SIMATS) Chennai 602105 Tamil Nadu India pepsiva9@gmail.com; Department of Physics, CMR Institute of Technology Bengaluru 560037 Karnataka India balan.ramdas@gmail.com; Department of Chemistry, MVJ College of Engineering Bengaluru 560067 Karnataka India; CoE Materials Science/Sensors & Nanoelectronics, Department of Chemistry, CMR Institute of Technology Bengaluru 560037 Karnataka India; VTU-Research Centre Affiliated to Visvesvaraya Technological University Belagavi 590018 Karnataka India; Department of Chemistry, Periyar University Salem 636011 Tamilnadu India; PG and Research Department of Chemistry, Pachaiyappas College, University of Madras Chennai 600030 Tamilnadu India; Department of Environmental Engineering, Faculty of Engineering, King Mongkut's University of Technology Thonburi Bangkok 10140 Thailand; Department of Materials Engineering, Indian Institute of Science Bengaluru 560012 India; Department of Chemistry, Faculty of Science, Chulalongkorn University Pathumwan Bangkok 10330 Thailand

## Abstract

In the present study, the n-SnO_2_/p-CuFe_2_O_4_ (p-CFO) complex was prepared by a two-step process. p-CFO synthesized by the molten salt method was coated with SnO_2_ synthesized by a facile *in situ* chemical precipitation method. The formation of n-SnO_2_/p-CFO was confirmed by powder X-ray diffraction (PXRD). Scanning electron microscopy (SEM) images showed that the sharp edges of uncoated pyramid-like p-CFO particles were covered by a thick layer of n-SnO_2_ on coated p-CFO particles. The complete absence of Cu and only 3 wt% Fe on the surface of the n–p complex observed in the elemental analysis using energy-dispersive X-ray spectroscopy (EDX) on the n–p complex confirmed the presence of a thick layer of SnO_2_ on the p-CFO surface. Diffuse reflectance spectroscopy (DRS) was employed to elucidate the bandgap engineering. The n-SnO_2_/p-CFO complex and p-CFO showed 87% and 58.7% methylene blue (MB) degradation in 120 min under sunlight, respectively. The efficiency of the n–p complex recovered after 5 cycles (73.5%) and was found to be higher than that of the uncoated p-CFO (58.7%). The magnetically separable property of the n–p complex was evaluated by using vibration sample magnetometry (VSM) measurements and it was confirmed that the prepared photocatalyst can be easily recovered using an external magnet. The study reveals that the prepared complex could be a potential candidate for efficient photodegradation of organic dyes under sunlight due to its efficient recovery and reusability owing to its magnetic properties.

## Abstract 2

The synthesis of n-SnO_2_/p-CuFe_2_O_4_ to degrade toxic methylene blue dye under natural sunlight and its mechanism.

## Graphical abstract

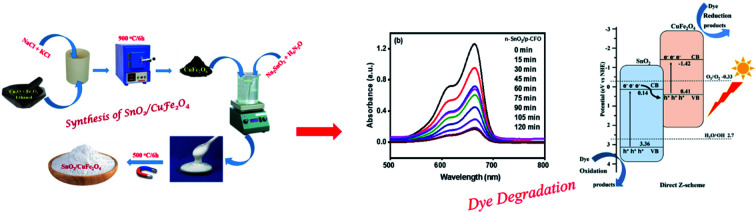

## 1 Introduction

The development of catalysts for the effective degradation of organic dye pollutants in wastewater is one of the promising research topics in the arena of environmental remediation. Among various organic dyes, methylene blue is a phenothiazine derivative that is highly toxic, carcinogenic, and predominant in industrial effluents which could cause serious health hazards upon intake.^1^ Traditional techniques such as ozonation, adsorption, *etc.* cannot eliminate the toxicity of these dyes due to various constraints.^2^ The development of photocatalysts for degradation of these dyes is one of the methods recently developed which uses direct solar energy as a source for effective degradation.^3^ Amidst different classes of materials, metal oxides such as TiO_2_ and ZnO are well-known semiconductor photocatalysts for dye degradation.^3–8^ Tin oxide (SnO_2_) is an n-type metal oxide well-studied photocatalyst for dye degradation owing to its superior optical, electrical, and electrochemical properties.^9–13^ It is a viable photocatalyst for practical applications due to facile production, low cost, eco-friendly, good chemical and biological inertness, high photosensitivity, and thermodynamic stability.^14,15^ Nevertheless, separating the photocatalyst from treated water and reuse is challenging, especially in the nano-form due to its high dispersive nature. In these cases, magnetic photocatalysts are advantageous owing to their ease of separation post usage. Therefore, magnetic spinel ferrites such as MFe_2_O_4_ (M = Cu, Co, Zn, Mn, Ni) have gained considerable attention.^16–18^ CuFe_2_O_4_ (CFO) is one of the important inverse spinel ferrite a p-type material possessing attractive magnetic, electronic, and optical properties; studied as a catalyst for a variety of applications including reduction,^19^ oxidation,^20^ photocatalytic hydrogen production,^21^ and photocatalytic degradation of dyes.^22–25^ Unfortunately, CFO has a low quantum efficiency due to the rapid recombination of photogenerated electron–hole pairs. This separation of the electron–hole pairs can be improved by transition metal graft or composite to form heterojunction or complex formation.^26–33^ Mostly, p–n type heterojunction of composites materials were reported to have effective photogenerated electrons/holes separation due to electric field created in the junction *in virtue* to enhance the photocatalytic activity.^34–36^ Few example for the p-type CFO utilized to decorated the diverse metal oxides and applicable to the various research field in recent universe; TiO_2_/CFO,^37,38^ RGO/CFO/TiO_2_,^39^ ZnO/CFO,^40^ CFO/PAMAM (polyaminodoamine dendrimers),^41^ CuFe_2_O_4_/Bi_4_Ti_3_O_12_.^27,31,42^ Limited work was reported for n-SnO_2_/p-CFO; it was used for sensing, optical, and enhancing the electrical properties of the sample.^43–45^ Up to the author's knowledge, there was no coherent application reported for the past decade. We have constructed the n–p type complex instead of the p–n type and used it for the environmental remediation of toxic dyes.

In the present work, the n-SnO_2_/p-CFO complex was successfully synthesized by a two-step process, first p-CFO microcrystals were prepared by the molten salt method, and secondly, *in situ* n-SnO_2_ was grown on p-CFO by chemical precipitation method. The photocatalytic activity was investigated for the prepared n-SnO_2_/p-CFO complex under natural sunlight for photodegradation of methylene blue (MB) dye. n-SnO_2_/p-CFO complex showed higher catalytic activity under direct sunlight than p-CFO due to the formation of the n–p complex. The magnetic property of the composite enables the easy recovery of the composite from the water body for reuse. To the best of our knowledge, there is no prior reported literature on the facile preparation of n-SnO_2_/p-CFO photocatalyst and its application in MB dye degradation under natural sunlight irradiation. The proposed charge separation mechanism was declared the photocatalytic degradation of organic dyes.

## 2 Experiment work

Analytical reagent (AR) graded chemicals were employed to develop the complex formation with the below experiments. The prepared compounds were characterized using techniques such as powder X-ray diffraction (PXRD, Bruker D2 phaser, at scan speed 0.5° min^−1^), scanning electron microscopy (SEM, ZEISS Ultra-55), diffuse reflectance spectrometry (DRS, PerkinElmer, lambda 365 spectrophotometer), Electrochemical workstation (CHI660E, CH Instruments), vibrating sample magnetometer (VSM, Lakeshore) at room temperature (27 °C, RT) for applied magnetic field ranges from −0.5 Tesla to +0.5 Tesla.

### 2.1 Preparation of p-CFO

The p-type CuFe_2_O_4_ (p-CFO) was prepared by the molten salt synthesis (MSS) method using Cu_2_O (Thomas Baker, India), Fe_2_O_3_ (Thomas Baker, India), NaCl (Thomas Baker, India), and KCl (Thomas Baker, India) chemicals. The stoichiometric ratio of 1 : 2 starting materials *i.e.*, 1.430 g of Cu_2_O and 3.139 g of Fe_2_O_3_ were ground in the agate pestle mortar in the ethanol medium for 1 hour. The dried mixture powder was put along with the eutectic mixture of the mediator, 5.727 g of NaCl and 7.604 g of KCl in a 100 ml capacity recrystallized alumina crucible and heat-treated at 900 °C for 6 h inside the muffle furnace and allowed furnace cool. The solidified molten salt was dissolved and washed with a copious quantity of deionized water to remove mediator alkali chloride salts. The residue black mass was dried in a hot air oven overnight.

### 2.2 Preparation of n-SnO_2_/p-CuFe_2_O_4_ complex

**Fig. 1:**
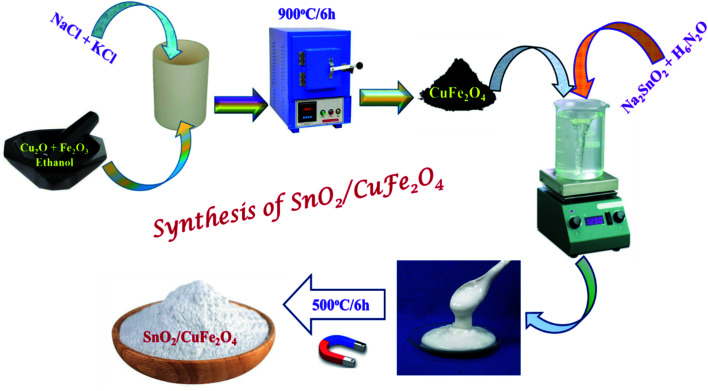
Schematic diagram represents the facile route to prepare the n-SnO_2_/p-CFO complex.

First, the Sodium stannate solution was prepared by dissolving 5 g of Na_2_SnO_3_·2H_2_O (SD fine chemicals) in 100 ml of distilled water and adding 5 ml of hydrazine hydrate (SD fine chemicals). Followed by 1 g of p-CFO microcrystals were added to the transparent sodium stannate solution and stirred for 1 h. The p-CFO mixed solution was kept undisturbed for the growth of n-SnO_2_ on p-CFO microcrystals, assisted with intermediate ultrasonication. The resultant white slurry was washed with copious distilled water, and later magnetically separated and dried at 80 °C overnight in an oven. Post drying, the sample was heat-treated at 500 °C for 6 h resulting in fine powder which was later used for further characterization and photocatalytic studies. The proposed schematic diagram was illustrated in Fig. 1.

### 2.3 Photocatalytic studies

The photocatalytic performance of the prepared catalysts was evaluated for MB dye degradation under sunlight exposure. In the present study, 100 mg of the prepared photocatalyst was suspended in 100 ml of 3 mg L^−1^ MB dye solution. The suspension was agitated at 200 rpm using a magnetic stirrer (REMI 5 ML) in dark conditions for 30 minutes to achieve dye adsorption–desorption equilibrium on the composite photocatalyst.^46,47^ Post adsorption–desorption equilibria, the suspension was positioned in an open place under direct sunlight between 11 a.m. to 1 p.m. as per Indian Standard Time (IST). During this process, periodically 5 ml of suspension were extracted and the solution was recorded using a UV-Vis spectrophotometer to quantify the MB dye content by measuring absorbance at 663 nm. The used photocatalyst was recovered from the treated MB solution with the aid of a magnet (magnetic strength = 0.3 Tesla), washed with distilled water, and dried at 100 °C overnight. The photocatalytic experiments were repeated in the same conditions using a recovered catalyst to check the reusability. Scavenger test was performed maintaining same photocatalytic experiment condition with the addition of scavengers such as benzoquinone (BQ), potassium iodine (KI), potassium bromate (KBrO_3_), and isopropanol (IPA) for effective charge separation (e^−^/h^+^) provide enormous radicals; such as superoxide and hydroxyl radical respectively.

The efficiency of the dye degradation was calculated using the expression

1 % degradation = (*C*_0_ − *C*_*t*_)/*C*_0_ × 100

where *C*_0_ is the initial absorbance, and *C*_*t*_ is the absorbance at time *t*.

## 3 Results and discussion

### 3.1 Characterization

#### 3.1.1 XRD studies

**Fig. 2:**
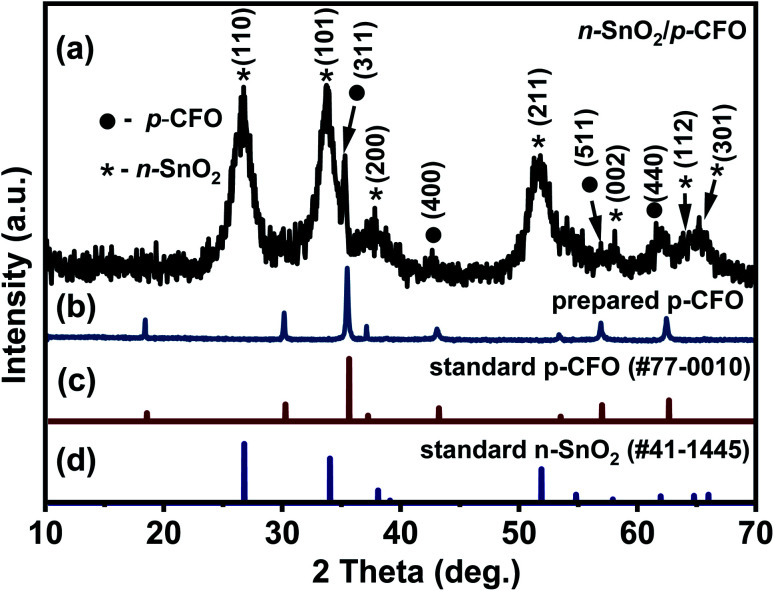
Shows the XRD pattern of (a) prepared n-SnO_2_/p-CFO complex, (b) prepared p-CFO, (c) standard p-CFO pattern, and (d) standard n-SnO_2_ pattern.

The XRD pattern of the core compound prepared by MSS using a eutectic mixture of NaCl–KCl mediated salts is depicted in Fig. 2b. The pattern is consistent with the standard data of p-CFO (Fig. 2c), confirming the formation of the pure cubic-p-CFO phase. The diffraction pattern of the n-SnO_2_/p-CFO complex is shown in Fig. 2a. The diffraction peaks detected at 26.64°, 33.88°,51.8°, 57.82°, 64.92°, and 66.02° are consistent with n-SnO_2_ of tetragonal structure P42mnm space group (136); the standard SnO_2_ data shown in Fig. 1d.^48^ The other four peaks observed at 35.52°, 42.13°, 57.1°, and 62.74° attribute to cubic-p-CFO diffraction planes (311), (400), (511), and (440), respectively.^49^ The peak intensity of the p-CFO is relatively weak due to the *in situ* deposition of n-SnO_2_ on the p-CFO. The peak profile of n-SnO_2_ was observed to be broadened, which affirms that it is in nano-crystalline form.

#### 3.1.2 Scanning electron microscope studies

**Fig. 3:**
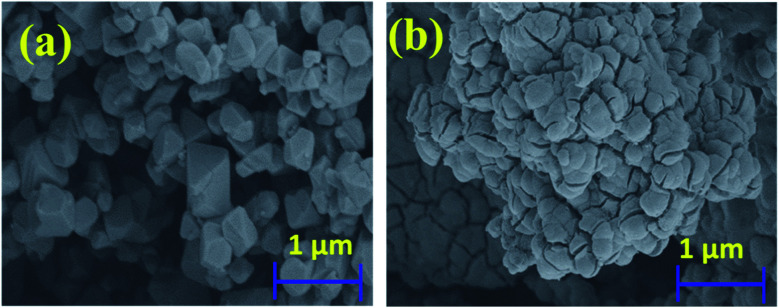
SEM image of (a) p-CFO and (b) n-SnO_2_/p-CFO complex.

Fig. 3 shows the SEM images of the p-CFO and p-CFO coated with n-SnO_2_. Uncoated p-CFO particles are micron size pyramidal shape particles with sharp edges as shown in Fig. 3a. p-CFO particles coated with n-SnO_2_ revealed smooth surfaces indicating that the sharp edges of p-CFO are covered by a thick layer of n-SnO_2_ as shown in Fig. 3b. From the EDX pattern of n-SnO_2_/p-CFO shown in the ESI (Fig. S1†) it is observed that only 3 wt% of Fe was observed and Cu was completely absent, confirming the formation of a thick layer of n-SnO_2_ on p-CFO surface resulting in n–p complex.^44^

#### 3.1.3 Calculation of optical band gap

The Kubelka–Munk (K–M function, eqn (2))^50,51^ was used to find the bandgap of the prepared p-CFO and n-SnO_2_/p-CFO complex.

**Fig. 4:**
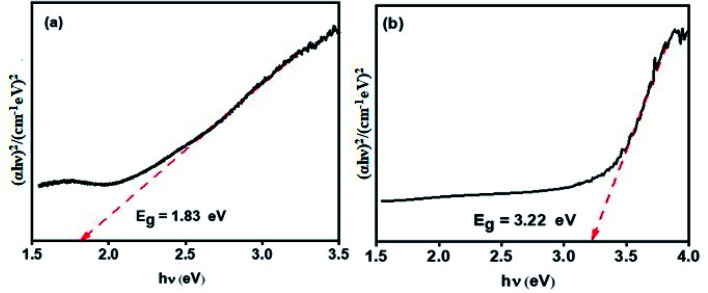
Kubelka–Munk plot for (a) p-CFO (narrow band gap) and, (b) n-SnO_2_/p-CFO (wide band gap).

2 (*αhν*)^*n*^ = *A*(*hν* − *E*_g_)

where *n* is determined from the type of optical transition of a semiconductor (*n* = 2 for direct transition and *n* = 1/2 for indirect transition), while *α*, *hν* and *E*_g_ are the absorption coefficient, the incident photon energy, and the bandgap energy, respectively; *A* is a constant. Fig. 4a and b show the K–M plot, *i.e.*, (*αhν*)^2^ plotted against the photon energy (*hν*) of p-CFO and n-SnO_2_/p-CFO complex respectively. From the plots, the energy bandgap was derived by taking tangent from the linear part of the curve intercepting the *x*-axis and the values found were 1.83 eV and 3.22 eV for p-CFO and n-SnO_2_/p-CFO respectively. The optical bandgap of the n-SnO_2_/p-CFO complex is more than p-CFO and less than that of the bulk n-SnO_2_ (3.6 eV) attributing to the formation of complex structure. The optimum encapsulation of the bare sample can reduce the bandgap of the pristine materials.^52,53^ However, the bandgap is closer to the bulk n-SnO_2_, due to the dominant shell formation of SnO_2_ which is in agreement with XRD & SEM analysis. Additionally, the clear scheme for the band position and charge separation mechanism was designated in Fig. 7.

#### 3.1.4 Magnetic studies

**Fig. 5:**
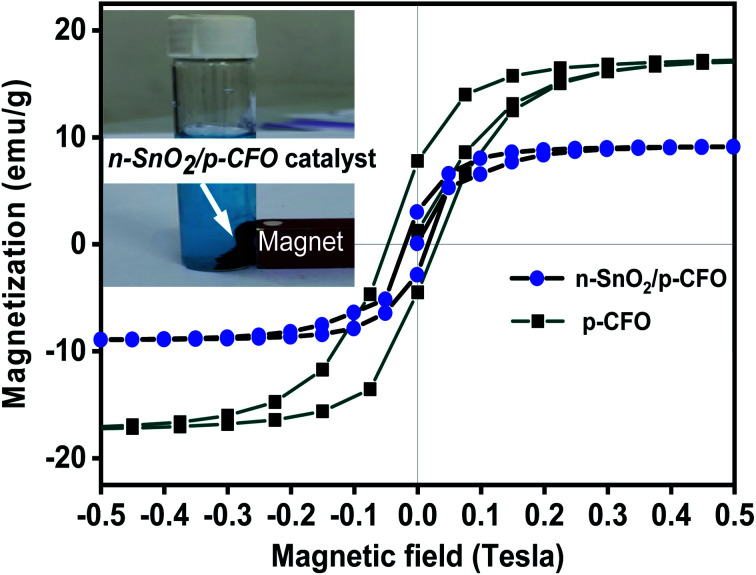
Hysteresis curves of the prepared p-CFO and n-SnO_2_/p-CFO complex.

Fig. 5 depicts the hysteresis curves of the prepared p-CFO and n-SnO_2_/p-CFO complex. The VSM measurement was carried out at room temperature for magnetic field range from −0.5 to +0.5 Tesla. The saturation magnetization (Ms) values for p-CFO and n-SnO_2_/p-CFO were determined to be 17.44 emu g^−1^ and 8.9965 emu g^−1^, respectively. *M*_s_ value generally implies the ease with which powder can be recovered with an external magnetic field. The coercivity (*H*_c_) and retentivity (*M*_R_) of the n-SnO_2_/p-CFO composite are 0.017 Tesla and 2.90 emu g^−1^ respectively. These values are diminution compared to p-CFO, which is 0.038 Tesla and *M*_R_ = 6.13 emu g^−1^. This is due to the presence of the nonmagnetic compound SnO_2_ in the complex. However, the magnetic characteristics of the resulting complex are sufficient to separate the composite magnetically post photocatalytic process which is highly recommended for recovery and reusability for sustainable utility (illustrated in inset Fig. 5).

### 3.2 Photocatalytic degradation evaluation

**Fig. 6:**
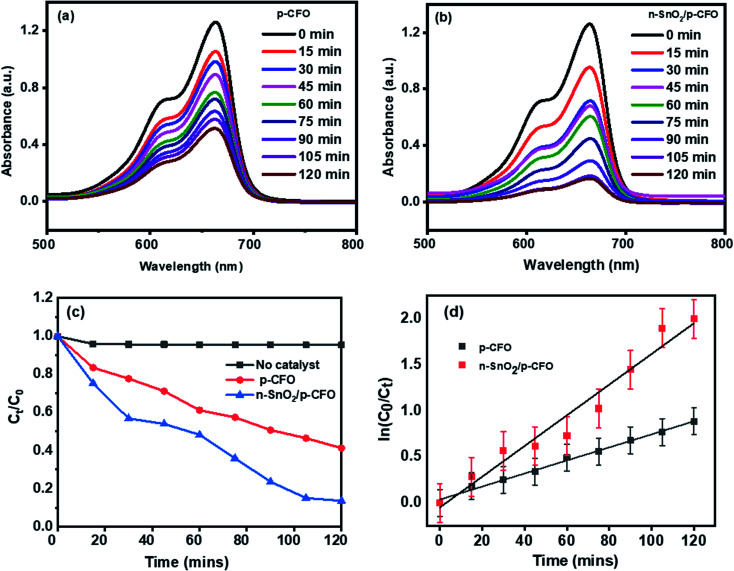
Photocatalytic MB dye degradation under direct sunlight irradiation (a) p-CFO, (b) n-SnO_2_/p-CFO, (c) *C*_*t*_/*C*_0_ plot, (d) ln *C*_0_/*C*_*t*_*vs.* time plot for the determination of rate constant.

Fig. 6a and b shows the absorption spectrum of MB dye drawn during the photocatalysis under sunlight by p-CFO and n-SnO_2_/p-CFO catalyst respectively. It is observed that the intensity of the absorption peak at 663 nm gradually decreased concerning catalytic time. The photocatalytic degradation efficiency plot (*C*_*t*_/*C*_0_*vs.* time) of the studied catalysts is shown in Fig. 6c. The maximum MB degradation of the p-CFO and n-SnO_2_/p-CFO photocatalysts were observed 58.7% and 87% respectively at 120 min which revealed superior photocatalytic activity of n-SnO_2_/p-CFO photocatalyst. Photodegradation of MB dye without the presence of catalyst conducted in the sunlight showed less than 5% degradation of the dye, which indicates the efficiency of the prepared photocatalyst.

**Fig. 7:**
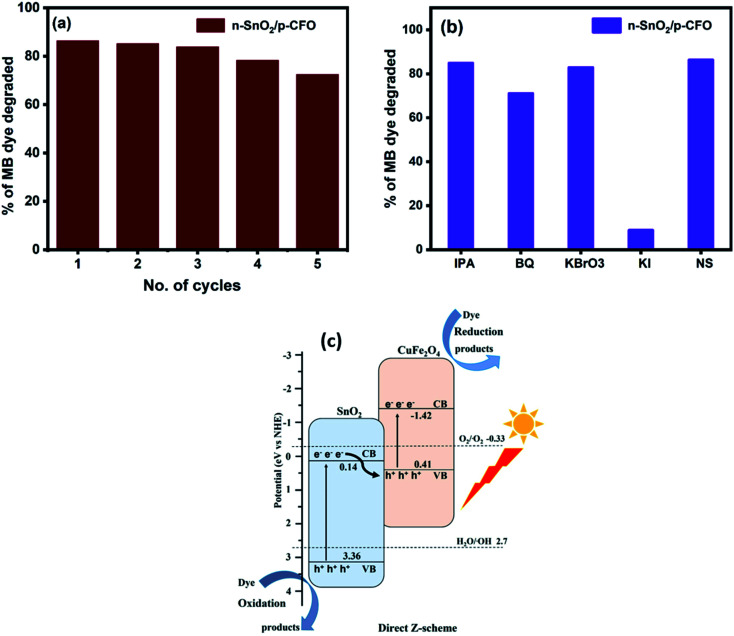
Depicts the photocatalytic MB degradation for (a) reusability (b) degradation percentage for various samples and (c) indicates the energy band scheme for n-SnO_2_/p-CFO complex.

The photodegradation of dyes usually follows pseudo-first-order kinetics and we analyzed this behavior for our reaction studies.3
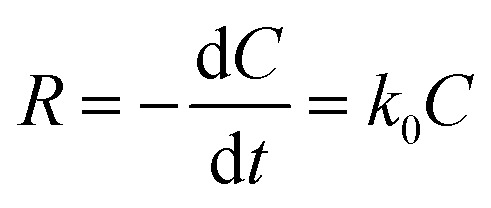
4
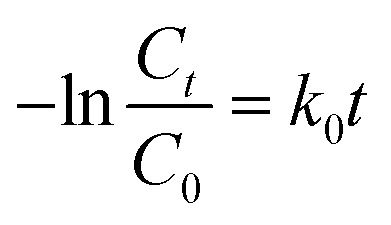
where *C*_0_ and *C*_*t*_ denoted the initial dye solution concentration and concentration at the time respectively. The plot of ln(*C*_0_/*C*_*t*_) *versus* time for all photocatalysis with 3 ppm dye concentration and 100 mg/100 ml catalyst concentration was observed as a linear plot with a correlation coefficient (*R*^2^) of 0.95–0.99 confirming their pseudo-first-order kinetics. The n-SnO_2_/p-CFO complex showed the steepest slope for the photodegradation kinetics as shown in Fig. 6d implying the high catalytic ability. The relevant parameters of photodegradation kinetics for different photocatalysts are shown in Table 1. The photocatalytic activities of TiO_2_, ZnO, MFe_2_O_4_ (M = Mn, Cu, Ni, Co, Zn) were presented in a Table 2 for comparison.

**Table 1:** Kinetic rate constant for p-CFO and n-SnO_2_/p-CFO complex

Compound prepared	Rate constant (K min^−1^)	Correlation coefficient (*R*^2^)
CFO	0.00708	0.9937
n-SnO_2_/p-CFO	0.01665	0.9615

**Table 2:** Comparison of photocatalytic/catalytic activity of ZnO, TiO_2_, SnO_2_, and MFe_2_O_4_^a^

S. No	Material	Dye	Degradation (%)	Irradiation source	Time (min)	Reference
1	ZnO	MB	93			8
2	TiO_2_	MB	66	UV (*λ* = 254 nm)	180	3
3	ZnO	MB, MO	Degradation rate is proportional to UV intensity	UV (*λ* = 365 nm)		5
4	Calcined abalone shell with 23.4% TiO_2_ loading	MB	100	Natural sunlight	140	7
5	SnO_2_	MB	100	UV (*λ* = 365 nm)	70	11
6	SnO_2_	Congo red	90	UV (*λ* = 365 nm)		13
	TiO_2_ degussa P-25		88			
7	SnO_2_	MB	3.8 time better activity than bulk SnO_2_	UV (*λ* = 365 nm)		12
8	SnO_2_	RhB	92	UV (*λ* = 365 nm)	120	10
9	SnO_2_	MB	98.5	Natural sunlight	80	51
10	SnO_2_	RB	99.3	Natural sunlight	180	15
		MB	96.8		240	
11	ZnFe_2_O_4_	RhB	98	UV (*λ* = 365 nm)	120	15
12	MnFe_2_O_4_	Direct red 81 dye	56.5	Natural sunlight	120	16
13	CuFe_2_O_4_	Acidic orange	87.6	Catalyst for reduction of organic compounds		18
14	MFe_2_O_4_ spinel (M = Cu, Ni, Co, Zn)		100	Catalytic reduction of 4-nitrophenol		19
15	Core–shell carbon dot@MFe_2_O_4_ (M = Mn, Zn and Cu)		>95	Catalytic reduction of *p*-nitropheno		25
18	p-CuFe_2_O_4_	MB	58.7	Natural sunlight	120 min	This work
19	n-SnO_2_/p-CuFe_2_O_4_	MB	87	Natural sunlight	120 min	This work

a MB: methylene blue; MO: methyl orange; RB: rose bengal; RhB: rhodamine B.

#### 3.2.1 Reusability and scavengers studies of the photocatalyst

The recovered n-SnO_2_/p-CFO complex catalyst was studied for recyclability for two cycles and the results are depicted in Fig. 7a. The results represented consecutive cycles with 85.2%, 83.9%, 78.3% and 73.5% efficiency suggesting excellent reusability of the prepared catalyst. The XRD pattern of the used catalyst after 5 cycles is shown in ESI, Fig. S2,† it depicts decrement in the intensity of the surface coated SnO_2_ peaks due to which the efficiency is droped to 73.5%. The scavenger test was carried out for n-SnO_2_/p-CFO to identify the reactive species involved in this photocatalysis mechanism by the addition of scavengers such as benzoquinone (BQ), potassium iodide (KI), potassium bromate (KBrO_3_), and isopropanol IPA for ˙O_2_, h^+^, e^−^ and ˙OH respectively. The scavenger test plot of MB degradation percentage was calculated with and without scavengers for n-SnO_2_/p-CFO complex is shown in Fig. 7b. The degradation efficiencies were greatly prevented by KI (77.5%), and a meager decrease by the addition of KBrO_3_ (3.5%), IPA (1.5%), and BQ (15.3%). Thus, these result of the trapping experiments under the sunlight demonstrates the photogenerated holes (h^+^) are the main active species triggering the photocatalytic degradation reaction to take place on the surface of the photocatalyst.

#### 3.2.2 Plausible mechanism of photodegradation

Photocatalyst constitutes tetragonal-SnO_2_ (n-type semiconductor) and cubic-CuFe_2_O_4_ (p-type semiconductor) semiconductors having band gap of 3.22 eV and 1.83 eV respectively. SEM micrograph and optical band gap of the SnO_2_/CFO confirms that SnO_2_ is completely coated on CFO. Hence, the thick SnO_2_ coating forbids maximum light reaching the inner core c-CFO. Therefore, SnO_2_/CFO suspension solution under sunlight promotes the formation of electrons in the conduction band (CB,e_CB_^−^) and holes in the valence band (VB,h_VB_^+^) of the SnO_2_ (eqn (5)).

5 SnO_2_ + *hν* → e_CB_^−^ + h_VB_^+^

The potential values of EVB and ECB of the semiconductors dictates reduction and oxidation of the photogenerated electron – holes in the degradation process.^30,31^ It was calculated using eqn (6) and (7).

6 ECB = *χ* − *E*^e^ − 0.5*E*_g_

7 EVB = ECB + *E*_g_

where *E*_g_ is the optical band gap calculated from the K–M plot, *E*^e^ is the energy of free electrons on the NHE scale factor (*i.e.*, 4.50 eV), and *χ* is the absolute electronegativity of the semiconductor. The calculated values of the ECB and EVB for c-CFO are −1.42 eV and 0.41 eV, and for SnO_2_ coated on the composite, the values are 0.14 eV and 3.36 eV. The band diagram is shown in the Fig. 6c. The potential ECB of the CFO in SnO_2_/CFO is more negative than the reduction potential of O_2_/˙O_2_ (−0.33 eV vs. NHE) and the EVB of SnO_2_/CFO is more positive than the oxidation potential of H_2_O/˙OH (+2.7 eV vs. NHE). Moreover, coupling of two different types of semiconductors forms a p–n junction and the photoexcited electrons from the CB of SnO_2_ combines with the holes generated from CFO by the driving force due to the inner electric field and could be attributed to Z-scheme mechanism.^32^ Hence, the photogenerated electrons in the higher CB edge of CFO and the higher VB edge of SnO_2_ could take part in the reduction and oxidation reaction of the MB dye. In addition, scavenger test confirmed the role of holes as major reactive species which paved the evident path to Z-scheme mechanism. During the scavenger test post addition of KI scavenger for holes there was dramatic decrease in the % dye removal suggesting holes were the dominant active species, BQ had considerable effect and IPA had weak effect implying superoxide radicals and hydroxyl radicals are also vital to elucidate the Z-scheme mechanism. The flow of electrons was further confirmed by the interface formation of p–n junction using EIS spectroscopy by varying the frequency from 1–106 Hz with standard 3-electrode set-up. The Nyquist plot showed in ESI, Fig. S3† represents two semi circles. One with a lager diameter which corresponds to c-CFO and the other smaller one due to the SnO_2_/CFO composite. The decrement in the semi-circle in case of the composite indicates the fast flow of electrons compared to the parent material suggesting the formation of a localized p–n junction interface which is assisting the easy passage of photoelectrons generated on the surface of the shell SnO_2_. The separated photogenerated holes in the valence band (h_VB_^+^) on the SnO_2_ will oxidize MB dye molecules directly due to their strong oxidizing ability (eqn (8)).^33^

8 h_VB_^+^ + MB → MB^+^˙

The n–p complex got better charge transport; the reaction mechanism was elaborately discussed in the below box.

## 4 Conclusions

Photocatalyst n-SnO_2_/p-CFO complex synthesized *via* a two-step process *i.e.*, molten salt synthesis of p-CFO followed by n-SnO_2_ by chemical precipitation method was characterized by powder XRD, SEM, EDX, and DRS. MB dye degradation studies under sunlight confirmed that the n–p complex is a more efficient photocatalyst (87%) than the uncoated p-CFO (58.7%) with better recovery and reusability properties and follows pseudo first order kinetics. Decrease in efficiency of the recovered photocatalyst after 5 cycles (73.5%) is due to loss of SnO_2_ from the surface of n–p complex as evidenced from the powder XRD patterns of the recovered n–p complex. This indicates that the n-SnO_2_ plays a major role in photocatalytic activity and p-CFO helps in easy recovery of the photocatalyst by magnetic separation as evidenced in VSM measurements. The scavenger and EIS studies revealed the role of photogenerated holes in the complex structure forming a localized n–p junction at the interface by the synergetic effect of n-SnO_2_ and p-CFO thereby preventing the recombination process. This study depicts the use of magnetically separable n–p complex n-SnO_2_/p-CFO as a potential catalyst candidate for the photodegradation of organic dyes under sunlight.

## Author contributions

Kaijiao Duan: methodology, investigation, reviewing and editing; Tingting Que: methodology, investigation, reviewing and editing; Sivasankar Koppala: conceptualization, methodology, investigation, project administration, writing – original draft preparation, supervision; Ramdas Balan: conceptualization, data curation, investigation, writing – original draft preparation, supervision; Budigi Lokesh: investigation, writing – original draft preparation; resources; Rahul Pillai: formal analysis, writing – reviewing; Selvaraj David: reviewing and editing; Parasuraman Karthikeyan: reviewing and editing; S. Ramamoorthy: resources; I. C. Lekshmi: resources; Patiya Kemacheevakul: reviewing and editing; Nagarajan Padmavathy: resources; Sathishkumar Munusamy: reviewing and editing.

## Conflicts of interest

There are no conflicts to declare.

## Supplementary Material

RA-012-D2RA01690G-s001
