# Supplementary material for: A facile route to synthesize n-SnO2/p-CuFe2O4 to rapidly degrade toxic methylene blue dye under natural sunlight
Source: RSC Adv. 2022 Jun 6;12(26):16544–53. doi: 10.1039/d2ra01690g (PMC9169069; doi:10.1039/d2ra01690g)
Supplement: RA-012-D2RA01690G-s001 [file RA-012-D2RA01690G-s001.pdf]

Electronic Supplementary Material (ESI) for RSC Advances.
This journal is © The Royal Society of Chemistry 2022

A facile route to synthesize n-SnO_2/p-$CuFe_2O_4$ complex to rapidly degrade the toxic Methylene Blue dye under natural sunlight

Kaijiao Duan[1], Tingting Que[1], Sivasankar Koppala[2*], Ramdas Balan[3*], Budigi Lokesh[4], Rahul Pillai[5,6], Selvaraj David[7], Parasuraman Karthikeyan[8], Sangeetha Ramamoorthy[5,6], I C Lekshmi[5], Patiya Kemacheevakul[9], Nagarajan Padmavathy[10], Sathishkumar Munusamy[11]

[1]School of Chemistry and Environment, Yunnan Minzu University, Kunming 650505, Yunnan, China

[2]Saveetha School of Engineering, Saveetha Institute of Medical and Technical Sciences (SIMATS), Chennai 602105, Tamil Nadu, India

[3]Department of Physics, CMR Institute of Technology, Bengaluru 560037, Karnataka, India

[4]Department of Chemistry, MVJ College of Engineering, Bengaluru 560067, Karnataka, India

[5]CoE Materials Science/Sensors & Nanoelectronics, Department of Chemistry, CMR Institute of Technology, Bengaluru 560037, Karnataka, India

[6]VTU-Research Centre affiliated to Visvesvaraya Technological University, Belagavi 590018, Karnataka, India

[7]Department of Chemistry, Periyar University, Salem 636011, Tamilnadu, India

[8]PG and Research Department of Chemistry, Pachaiyappas College, University of Madras, Chennai 600030, Tamilnadu, India

[9]Department of Environmental Engineering, Faculty of Engineering, King Mongkut's University of Technology Thonburi, Bangkok 10140, Thailand

[10]Department of Materials Engineering, Indian Institute of Science, Bengaluru 560012, India

[11]Department of Chemistry, Faculty of Science, Chulalongkorn University, Pathumwan, 10330, Bangkok, Thailand

*Corresponding Author: Sivasankar Koppala (pepsiva9@gmail.com) and Ramdas Balan (balan.ramdas@gmail.com)

Electronic supporting information

List of Figures

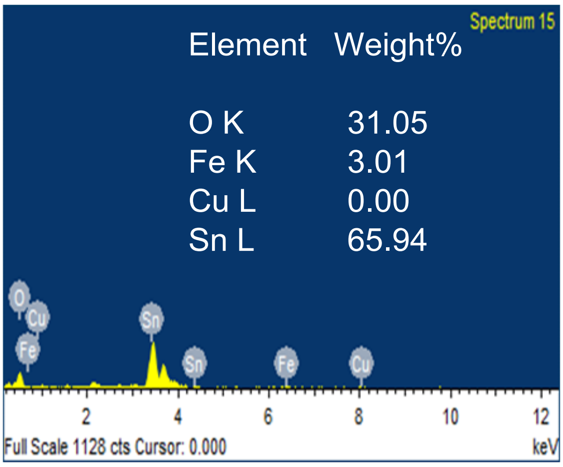

Fig.S1. EDX spectrum of n-SnO_2/p-CFO

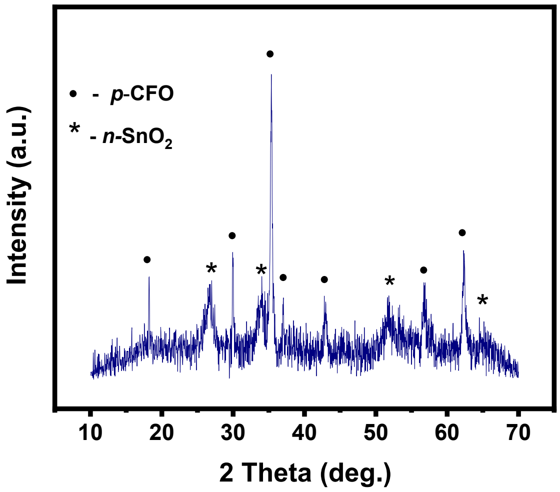

Fig.S2. XRD pattern of the used catalyst after 5 cycles

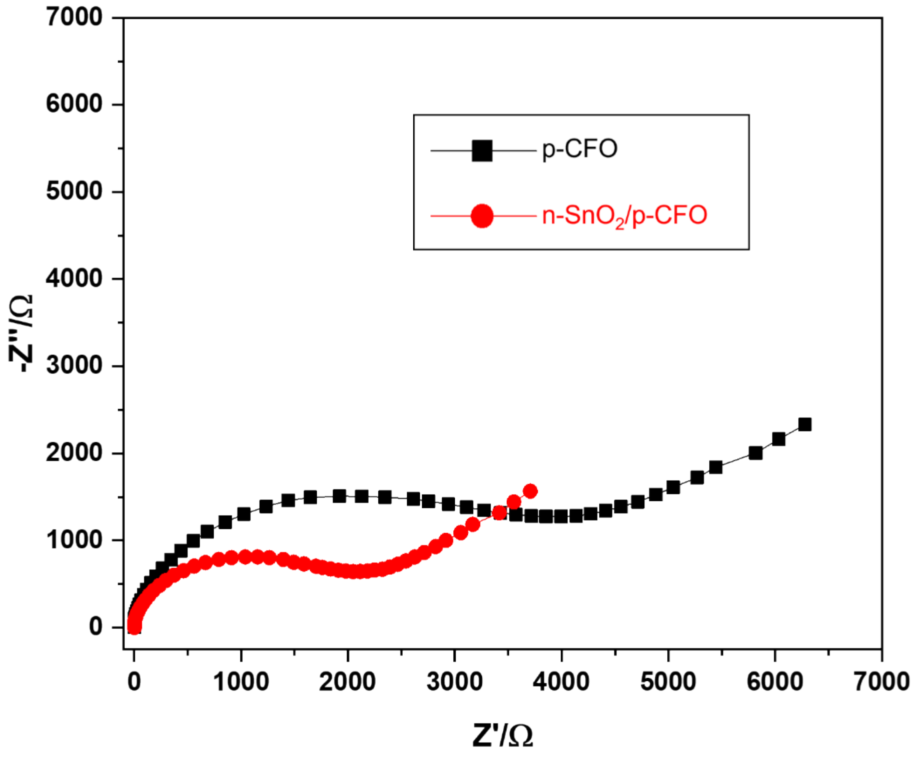

Fig. S3. Electrochemical Impedance spectroscopy (EIS) to probe the charge-transfer process of (a) p-CFO and (b) n-SnO_2/p-CFO
